# Olfactory LOVER: behavioral and neural correlates of autobiographical odor memory

**DOI:** 10.3389/fpsyg.2014.00312

**Published:** 2014-04-11

**Authors:** Maria Larsson, Johan Willander, Kristina Karlsson, Artin Arshamian

**Affiliations:** ^1^Gösta Ekman Laboratory, Department of Psychology, Stockholm UniversityStockholm, Sweden; ^2^Department of Psychology, Stockholm UniversityStockholm, Sweden

**Keywords:** autobiographical memory, odor, smell, neuroanatomy, experience, applied psychology

## Abstract

Autobiographical memories (AMs) are personally experienced events that may be localized in time and space. In the present work we present an overview targeting memories evoked by the sense of smell. Overall, research indicates that autobiographical odor memory is different than memories evoked by our primary sensory systems; sight, and hearing. Here, observed differences from a behavioral and neuroanatomical perspective are presented. The key features of an olfactory evoked AM may be referred to the LOVER acronym-**L**imbic, **O**ld, **V**ivid, **E**motional, and **R**are.

Autobiographical memories (AMs) are personally experienced events that may be localized in time and space (Conway and Pleydell-Pearce, 2000). In general, knowledge regarding AM function is well documented, although most of the evidence is based on recollections following a verbal cuing. However, during the past decade a number of studies have targeted memories cued by the sense of smell (e.g., Chu and Downes, 2000; Larsson and Willander, 2009; Zucco et al., 2012). The bulk of this research indicates that olfactory evoked AM differ from memories evoked by our primary senses; sight, and hearing. In particular, odor-evoked AM are older, more emotional, vivid, and relatively rare. The main aim of the present paper is to provide an overview regarding the observed differences from a behavioral and neuroanatomical perspective and to discuss potential applications of this knowledge. Also, the key features of an olfactory evoked AM – **L**imbic, **O**ld, **V**ivid, **E**motional, and **R**are are referred to the acronym LOVER.

## RETRIEVAL MODES IN AUTOBIOGRAPHICAL MEMORY

Autobiographical memories may be assessed differently depending on the research question. The most common method is the Galton-Crovitz method where individuals are given unimodal cues (e.g., words, pictures, or sounds) and asked to retrieve an AM for each cue (Crovitz and Schiffman, 1974). With successful retrieval, a short description of the event is provided along with ratings of experiential factors (e.g., vividness of the evoked memory, emotionality) of the recollected event. Typically, when all cues have been presented, the participant is asked to go back to each evoked event and date it (i.e., to indicate the age-at-event).

Evidence suggests that different retrieval strategies influence event selection and the age distribution of events (Conway and Pleydell-Pearce, 2000). Two modes of retrieval have been suggested: generative or direct (Moscovitch, 1995; Conway and Pleydell-Pearce, 2000; Conway, 2005). In generative retrieval, autobiographical information is validated in relation to an event description and the search process is intentional, iterative, and elaborative. In contrast, in direct retrieval, a cue activates a pattern of highly associated autobiographical information, resulting in an immediate and effortless recollection. Thus, selection is bypassed in the direct retrieval mode. It has been suggested that highly perceptual cues (e.g., odors) more often result in a direct recollection, whereas verbal information activate generative search strategies. Recent work has highlighted the functional neuroanatomy of direct and search oriented retrieval modes for autobiographical olfactory memories cued by odors and words (Arshamian et al., 2013). This study documented that both verbal and olfactory cues activated brain areas typically associated with retrieval of AM in general by recruiting prefrontal regions (e.g., dorsolateral prefrontal cortex), medial temporal lobe regions (e.g., parahippocampus), superior and middle temporal areas, fusiform gyrus, occipital areas, and the cerebellum (for reviews see, Svoboda et al., 2006; Cabeza and St Jacques, 2007). However, as compared to olfactory cues, the verbal cuing resulted in a substantially extended prefrontal activity where the right anterior prefrontal cortex, bilateral dorsolateral prefrontal cortex, middle frontal gyrus activation, and the left inferior frontal gyrus were recruited. These activations most likely reflect an increment of strategic retrieval demands induced by verbal labels as compared to odor cues that mapped directly on the olfactory memory representation (Conway and Pleydell-Pearce, 2000). In a related vein, Willander and Larsson (2007) reported that also the age distribution of memories might be affected by retrieval strategy. Here, the AMs triggered by olfactory information was localized in an earlier bump location (i.e., in childhood years) that may reflect an immediate recollection that bypass the retrieval selection process, whereas additional semantic information on the same odor cues resulted in a bump spanning both childhood and young adult age years, that may reflect a stimulation of a generative search process (cf. Conway and Pleydell-Pearce, 2000).

## THE LOVER ACRONYM OF AUTOBIOGRAPHICAL ODOR MEMORY

**FIGURE 1:**
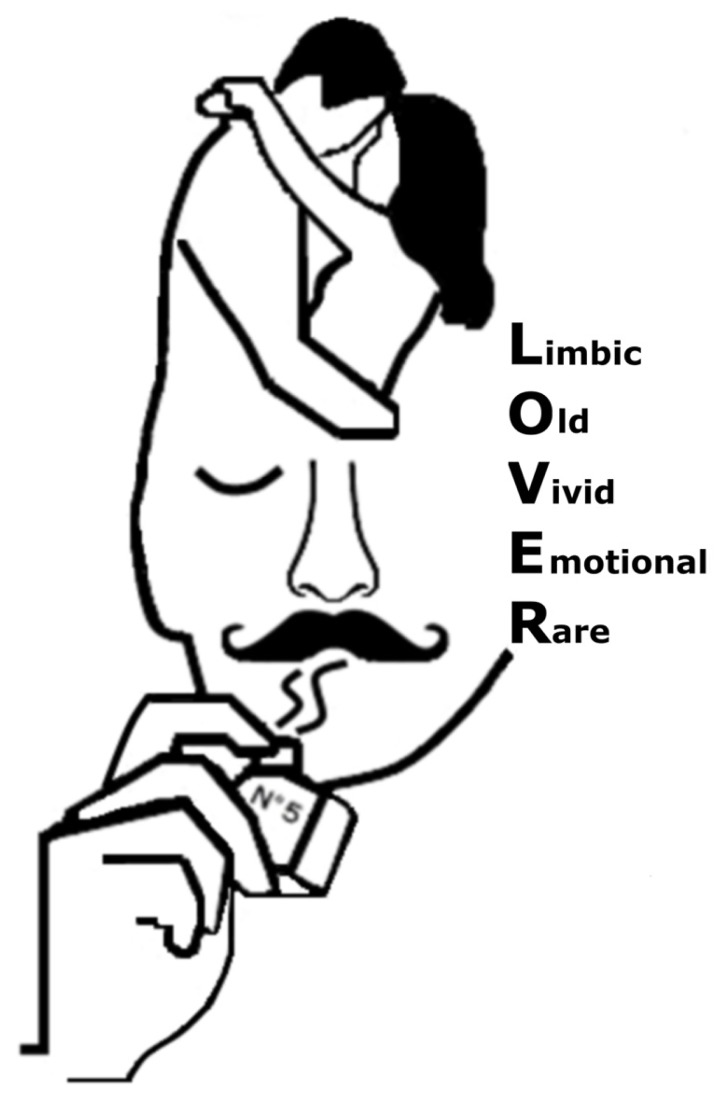
**Illustration of the acronym olfactory LOVER covering the core features of an autobiographical memory evoked by olfactory information**. Memories triggered by the sense of smell rely on the integrity of the **L**imbicsystem and are typically **O**ld, more **V**ivid, often **E**motional, and relatively **R**are as compared to autobiographical information evoked by our primary sensory systems.

As noted above, evidence shows that olfactory evoked personal information is different from information evoked by the primary senses. Below follows a description of the key features that differentiate odor-evoked AM from that triggered by other modalities. In the present work, these core features are referred to the acronym LOVER-**L**imbic, **O**ld, **V**ivid, **E**motional, and **R**are (see **Figure 1**).

### LIMBIC ACTIVATIONS

The sense of smell is characterized by a unique intimacy with the limbic system, where amygdala is located only one synapse away from the olfactory receptors. Moreover its extended neural network involves a large portion of the limbic and paralimbic cortices, including piriform cortex, amygdala and entorhinal cortices (Gottfried, 2010). In the first neuroimaging study of AM targeting odors, Herz et al. (2004) asked five participants whether they could recall a positive memory in which both the sight and scent of a perfume occurred. Later the participants were presented with the odors and pictures of the recollected perfumes in the fMRI while intentionally retrieving the memories. The results showed that odor cued memories were related to stronger activations in the amygdala and hippocampal regions than picture cued recollections. Arshamian et al. (2013) demonstrated that alongside amygdala and hippocampus, odor-evoked AMs also activated the limbic and paralimbic cortices of piriform cortex and entorhinal cortex and an extended limbic network (Morgane et al., 2005) including parahippocampus, insular cortex, and the orbitofrontal cortex.

### OLD MEMORIES

It is well documented that the age distribution of memories evoked by verbal information follows a distinct pattern involving three main components: the childhood amnesia, the bump, and recency. Childhood amnesia reflects the dramatic reduction of memories reported from early childhood. In contrast, a significantly larger number of memories are recalled from the ages of 10–30, a phenomenon that has been termed the bump. The third component, recency, reflects better retention of events occurring from the last years (Rubin, 1982). In the past decade, a number of studies have focused on the age distribution of odor-evoked memories. The overall results from these studies indicate that olfactory evoked autobiographical information is ontogenetically older than memories evoked by visual, auditory, and verbal information (Chu and Downes, 2002; Willander and Larsson, 2006, 2007; Willander et al., submitted). Specifically, the bump or the clustering of memories is localized to childhood that is the first decade of life (<10 years). Hence, distinct autobiographical episodes involving olfactory information are formed early in life than those comprising verbal and visual information. This observation supports research showing that associative odor learning begins very early in life, with events and experiences that may become accessible in old age through exposure to event-congruent olfactory information (Yeshurun et al., 2009). Targeting the neural correlates of olfactory evoked AM, Arshamian et al. (2013) investigated a group of adults with olfactory evoked AM. A comparison between evoked AMs from childhood (i.e., 3–10 years) and young adulthood (i.e., 11–20 years) revealed differences in brain activity. Specifically, odor memories derived from childhood were related to a stronger activity in the secondary olfactory cortex (i.e., orbitofrontal cortex), whereas olfactory evoked memories clustered in young adulthood were related to a more pronounced activity in the left inferior frontal gyrus, a brain region that supports semantic memory processing. Speculatively, it may be hypothesized that olfactory representations involved in the formation of AM initially may be more perceptually and imagery based, that with increasing age gradually shift to a more semantically driven consolidation.

### VIVID RECOLLECTIONS

Odor-evoked AM also differ with regard to phenomenology. A typical finding is that odor-evoked events are accompanied by stronger feelings of being brought back in time to the occurrence of the events (Herz et al., 2004; Willander and Larsson, 2006). Also, Chu and Downes (2002) highlighted that olfactory cued memories evoked more vivid and detailed memories than representations evoked by other sensory modalities. Targeting aversive memories, Toffolo et al. (2012) reported that odor-evoked memories of aversive events were more detailed than memories evoked by auditory but not visual cues. Interestingly, mimicking experiential evidence, also the functional neuroanatomy of olfactory AM indicate that brain areas involved in visual vividness such as occipital gyrus and precuneus are recruited during recollection, activation patterns that were more pronounced than for a verbal cuing (Arshamian et al., 2013). It is also worth noting that experiences of vividness have been linked to emotion such that high vividness is associated with increased emotionality (Todd et al., 2013). Hence, the heightened vividness experience in olfactory AM may relate to the typical emotional potency associated with odor-evoked memory recollection.

### EMOTIONAL EXPERIENCE

The olfactory sense is an emotional system (Lundström et al., 2010). Given that the olfactory nerves project directly to the amygdala complex, it has been proposed that odor-evoked AM are more emotional than memories cued by other modalities. Indeed, most studies suggest an emotional advantage of olfactory evoked AM over verbally and visually evoked memories (Herz and Cupchik, 1992; Herz, 1998; Larsson and Willander, 2009; but see Willander and Larsson, 2006; Toffolo et al., 2012; for different outcomes). In a recent study, Arshamian et al. (2013) explored the neural correlates of olfactory cued AM in an fMRI paradigm. The same odor-evoked memory was cued by either verbal or olfactory information. As compared to a verbal cue, an olfactory cued retrieval resulted in more activity in medial temporal lobe regions (e.g., parahippocampus, insula) and in the temporal poles. The latter activation is of particular interest as the temporal poles have been associated with positive memory processing (Piefke et al., 2003) that also was manifested among participants at the experiential level.

### RARE OCCURENCE

In anecdotes, it is often stated that odors act as common reminders of past experiences than other types of stimuli. However, a review of the empirical evidence indicates the opposite, namely that odor cues produce fewer memories and are associated with longer response latencies (Rubin et al., 1984; Goddard et al., 2005; Willander and Larsson, 2007; Willander et al., submitted). These findings suggest that odors may be less efficient reminders of past experiences than verbal or visual information. It has been proposed that cue specificity may underlie this discrepancy. Odors are more specific cues than verbal or pictorial information. As a consequence, odors will match fewer representations than more generic cues such as words or pictures. Indeed, research shows that if semantic information is provided with the odor cue (i.e., the odor identity) or when the odor is identified, more memories will be retrieved (Willander and Larsson, 2007; Yamamoto, 2008).

Relatedly, it is of interest to highlight that memories evoked by the olfactory sense in general have been thought about less often than memories evoked by other sensory cues (Rubin et al., 1984; Willander and Larsson, 2006). The implicit nature of olfactory representations and the low frequency of AMs probably underlie the experienced “suddenness” of an odor-evoked memory that may bias the notion of its powerfulness.

## UNIMODAL vs. MULTIMODAL CUING OF AUTOBIOGRAPHICAL ODOR MEMORIES

Almost all of the knowledge on odor-evoked AM is based on unimodal cuing, where an individual is presented to one odor and is subsequently asked to retrieve any personal associated information for that specific smell that may be defined in space and time. As noted, the results from this research indicate that odor-evoked AM are different from information triggered by verbal, visual, or auditory information. The observed differences are documented both at a behavioral and a neural level (e.g., Willander and Larsson, 2006; Arshamian et al., 2013; Karlsson et al., 2013; Willander et al., submitted).

A unimodal retrieval procedure (i.e., cues pertaining to one modality) entails that sensory information from different modalities is treated as separate entities rather than as a component of integrated multimodal representations. An important research question recently raised is therefore to determine the relative influence and hierarchy among modalities that are represented in a multimodal cue on the recollection of olfactory information (Karlsson et al., 2013; Willander et al., submitted).

Willander and Larsson (2007) indirectly addressed bimodal cues when individuals were asked to retrieve AM following single odors or odors presented in conjunction with their respective names. The results showed that semantic knowledge of an odor’s name affected the age distribution such that the memory peak in childhood observed for only odors was attenuated. Specifically, the peak took an intermediate position between the age distributions obtained for verbal cuing and odor cuing only. Also, semantic knowledge of the odors resulted in that the experiential factors (emotionality, brought back in time) mimicked a verbal cuing of AM. Hence, this outcome indicated that the age and phenomenology of memories vary with the number and types of cues available at retrieval.

In this vein, it is of interest to highlight results from a recent study that targeted multimodal retrieval of AM (Willander et al., submitted). Here, participants were randomized across three unimodal (pictures, sounds, odors) and one multimodal condition (picture + sound + odor). To maximize ecological validity, cues from the three unimodal conditions were presented simultaneously, whereas in the unimodal conditions cues were presented separately. The unimodal cues were selected so that they could be combined into a multimodal naturalistic context. For example, the context harbor was represented by a photo of a harbor by the sea containing fishing boats; sounds from fishing boats, sea birds, sea waves; and the smell of fresh fish. The results indicated that the number of olfactory evoked memories were fewer than the number of memories evoked by visually and multimodally presented cues. The unimodal cuing of AM replicated previous findings by showing a significant clustering of odor memories in childhood, and peaks of memories following visual and auditory cuing in young adulthood (e.g., Larsson and Willander, 2009). As noted, the analysis of the evoked memories following a multimodal cuing indicated a significant clustering of memories in young adulthood, mimicking that observed for our primary sensory systems. Also, modeling of the semantic content of the retrieved memories indicated that the multimodal content differed from odor-evoked content but not from visual content (Karlsson et al., 2013). Hence, these results suggest a hierarchy among modalities represented in multimodal cue information, and that the subordinate role that is played by the sense of smell may underlie the rare occurrence of odor-evoked AMs (Posner et al., 1976; Sinnett et al., 2007).

This outcome supports the notion of visual cue dominance in multimodal contexts. One important question in future research is to determine the role played by modality attention in multimodal settings.

## APPLIED POSSIBILITIES OF ODOR-EVOKED AUTOBIOGRAPHICAL INFORMATION

The literature on potential applications of olfactory AMs is scarce and portrays a mixed pattern of findings. Greenberg et al. (2011) examined whether odors could be used as memory cues to promote memory recollection in patients with semantic dementia. The results showed that odor cues were less effective reminders of past experiences than were verbal and visual cues. This was most likely a reflection of the early degeneration of anterior temporal regions in the dementia process, as the same regions also are fundamental for the integrity of the olfactory system. Other research has highlighted that autonomic functions are affected by AM. For example, Masaoka et al. (2012) demonstrated that odors that evoked AMs lowered the respiratory frequency as compared to odors that were unrelated to memory evocation. Likewise, Matsunaga et al. (2011) reported a decrease in heart rate, and an increase in skin-conductance following odor-evoked AMs. For example, Matsunaga et al. (2011) showed that immune responses associated with systemic inflammation could be inhibited by odor-evoked AMs. Further, Matsunaga et al. (2013) demonstrated that these immune responses were negatively correlated with activations in orbitofrontal cortex, precuneus, and the posterior cingulate cortex as determined by PET. This could indicate that inhibition of inflammatory mechanisms decrease as a function of the vividness and emotionality of the evoked memories (c.f. Arshamian et al., 2013).

Interestingly, individual differences in mood and personality traits have been found to interact with odor-evoked AM. For example, Masaoka et al. (2012) reported that participants who where high in trait anxiety experienced stronger feelings of being brought back in time to the occurrence of the event, and showed increments in arousal level during retrieval of odor-evoked AMs. Also, Matsunaga et al. (2011) reported that odor-evoked AMs that were associated with positive emotions increased positive mood states, such as comfort and happiness, and decreased negative mood states, such as anxiety. Moreover, Reid et al. (2014) studied experiences of nostalgia in the context of odors. They demonstrated that participants reported most nostalgia when the odors were arousing, familiar, and evoked AMs. Furthermore, odors that only evoked nostalgia induced more positive emotions than both non-nostalgic odors that evoked AMs, and those that did not. Participants that were generally more prone to nostalgia reported more odor-evoked nostalgia, but not more autobiographical events. Taken together, the research cited above suggests that olfactory evocation of autobiographical information has the potential to affect our autonomic functions and emotional state.

## AUTHOR CONTRIBUTIONS

Maria Larsson and Artin Arshamian jointly prepared the manuscript. Maria Larsson, Artin Arshamian, Johan Willander, and Kristina Karlsson wrote the manuscript. Artin Arshamian made the illustration.
